# Rare occurrence of pulmonary coinfection involving *Aspergillus fumigatus* and *Nocardia cyriacigeorgica* in immunocompetent patients based on NGS: A case report and literature review

**DOI:** 10.1097/MD.0000000000036692

**Published:** 2023-12-22

**Authors:** Jiaqing Ye, Yahua Li, Jiahao Hao, Minghui Song, Yumei Guo, Weili Gao, Cuiying Zheng, Yinqi Huang, Zhongjun Feng, Lijie Zhang

**Affiliations:** a The Third Hospital of Hebei Medical University, Shijiazhuang, Hebei, PR China; b Hebei Key Laboratory of Intractable Pathogens, Shijiazhuang Center for Disease Control and Prevention, Shijiazhuang, Hebei, PR China.

**Keywords:** *Aspergillus fumigatus*, immunocompetent patient, Next-Generation Sequencing (NGS), *Nocardia cyriacigeorgica*, pulmonary co-infection

## Abstract

**Rationale:**

In our search on PubMed, we found that reports of co-infections involving *Aspergillus fumigatus* and *Nocardia cyriacigeorgica* in the literature are notably scarce. Most cases have been documented in patients with compromised immune systems or underlying pulmonary conditions. In contrast, our patient did not present with any of these risk factors. Furthermore, there have been no recent incidents such as near-drowning or other accidents in the patient history. To the best of our knowledge, this case represents a hitherto unreported clinical scenario. To enhance comprehension, we conducted a comprehensive literature review by compiling a total of 20 case reports (spanning from 1984 to 2023) on co-infections involving Aspergillus and Nocardia species, retrieved from PubMed.

**Patient concerns and diagnosis:**

Chest CT revealed the presence of multiple nodules and clustered high-density shadows in both lungs. Bronchoscopy revealed mucosal congestion and edema in the apical segment of the right upper lobe of the lung, along with the presence of 2 spherical polypoid new organisms. The pathological analysis reported severe chronic inflammation with evidence of Aspergillus within the tissue. Next-Generation Sequencing of bronchoalveolar lavage fluid revealed the presence of reads corresponding to *A fumigatus* and *N cyriacigeorgica*. Positive cultures for *A fumigatus* and the Nocardia genus were yielded by prolonging the incubation of samples in the microbiology laboratory.

**Interventions:**

Treatment with voriconazole for *A fumigatus* and sulfamethoxazole-trimethoprim for *N cyriacigeorgica* infection was given.

**Outcomes:**

The patient improved and was discharged. After 6 months of telephone follow-up, the patient reported no clinical symptoms, discontinued the medication on his own.

**Lessons:**

*A fumigatus* and *N cyriacigeorgica* can manifest as a co-infection in immunocompetent patients. Clinicians should prioritize the significant advantages and value of NGS in detecting rare and mixed pathogens associated with pulmonary infections.

## 1. Introduction

*Aspergillus fumigatus* and *Nocardia cyriacigeorgica* are widely distributed in the natural environment, and both are opportunistic pathogens. Their spores or hyphae are inhaled through the human respiratory tract, invading the lungs and leading to pulmonary infections known as pulmonary aspergillosis (PA) and pulmonary nocardiosis (PN), respectively. The clinical symptoms and radiological presentations of pulmonary infectious with the pathogens lack specificity, rendering these types of cases prone to a misdiagnosis and underdiagnosis. Accurate differentiation necessitates laboratory techniques combined with clinical diagnostic progress to exclude all potential alternative diseases. A literature review revealed that cases of mixed infection involving these 2 pathogens are infrequent and are mostly observed in patients with compromised immune functions or underlying pulmonary conditions. In contrast, our patient did not present with any of these risk factors. Furthermore, there have been no recent incidents such as near-drowning or other accidents in the patient history. To the best of our knowledge, no similar cases have been reported before.

## 2. Case report

A 63-year-old male from Hebei, China, presented to the hospital 3 weeks prior to admission with fever, cough, and fatigue after exposure to cold temperatures. His highest recorded body temperature was 39.5°C, accompanied by weakness, night sweats, and chills. At the county hospital, the patient had received antibiotic treatment of ceftizoxime sodium and levofloxacin sodium treatment for 3 weeks following his initial symptoms. Although the temperature dropped to normal and the cough subsided, but chest CT imaging showed continuous progression. On September 19, 2022, he was admitted with a diagnosis of “pulmonary infection.” He worked as a security guard at a valve factory and was exposed to a humid environment with a mouldy odor. He had a smoking history of over 40 years (5 cigarettes/day). The patient had normal immune function, and he denied any history of COVID-19 infection, tuberculosis, corticosteroid use, diabetes, or surgical trauma.

Laboratory investigations revealed the following results: the patient exhibited a white blood cell count (WBC) of 4.79 × 10^9/L, falling within the reference range. However, the hemoglobin (HB) level was decreased to 109.10 g/L, and the erythrocyte sedimentation rate (ESR) displayed a markedly elevated value of 120.00 mm/h. Additionally, diminished levels were observed in plasma albumin (ALB) at 34.55 g/L and prealbumin (PA) at 184.1 mg/L. A chest CT scan (Day 3) revealed the presence of multiple nodules and clustered high-density shadows in both lungs. Cavity within multiple nodules in the upper lobe of the right lung. Enlarged mediastinal lymph nodes and localized pleural thickening were also noted. No significant abnormalities in other tests.

**Figure 1.**
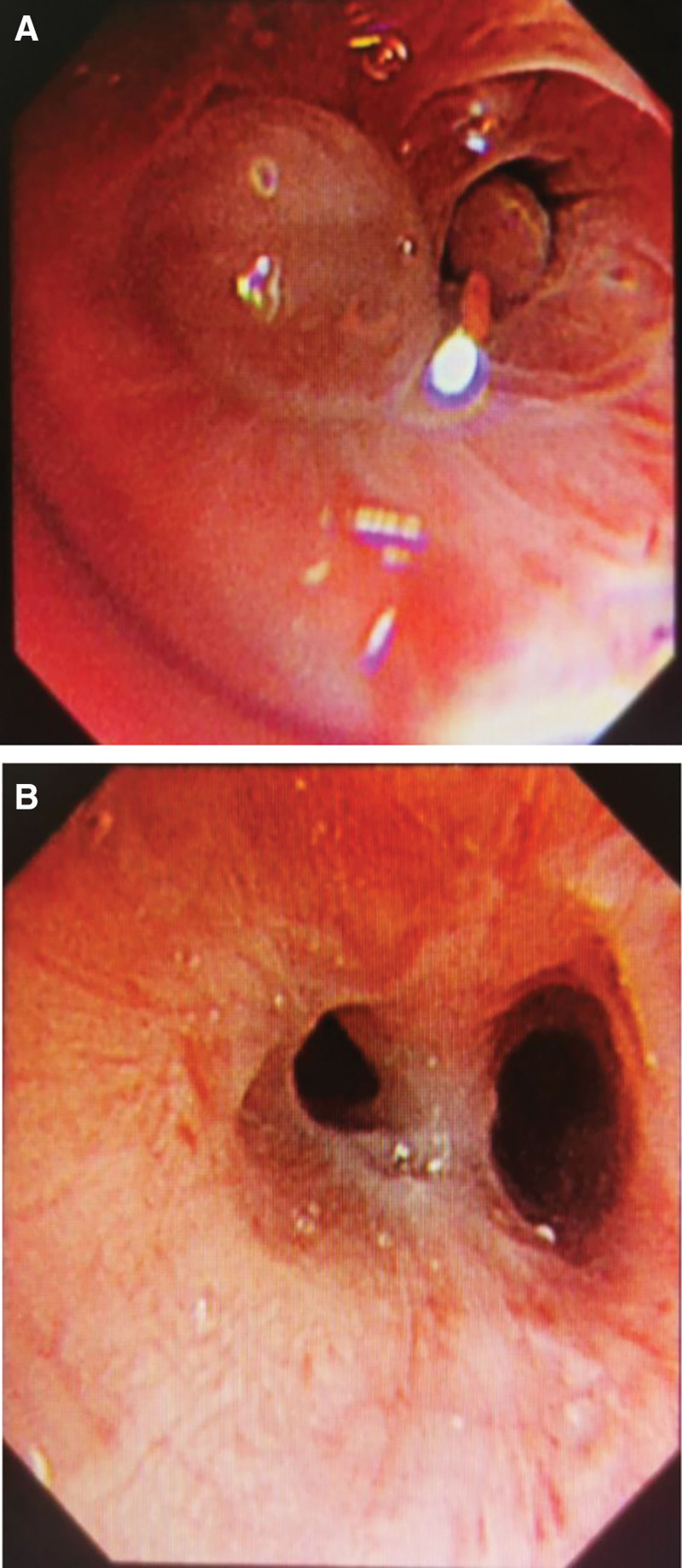
Electronic bronchoscopy examination (Day 5) revealed congestion and edema of the mucosa with the opening of the right upper lobe tip segment. Thread-like substances were observed covering the surface of the bronchial mucosa. Two spherical polypoid neoplasms were observed (A). Bronchoscopy examination (Day 22) showed reduced congestion and edema of the mucosa with the opening of the right upper lobe tip segment, and a decrease in the presence of thread-like material on the mucosal surface (B).

**Figure 2.**
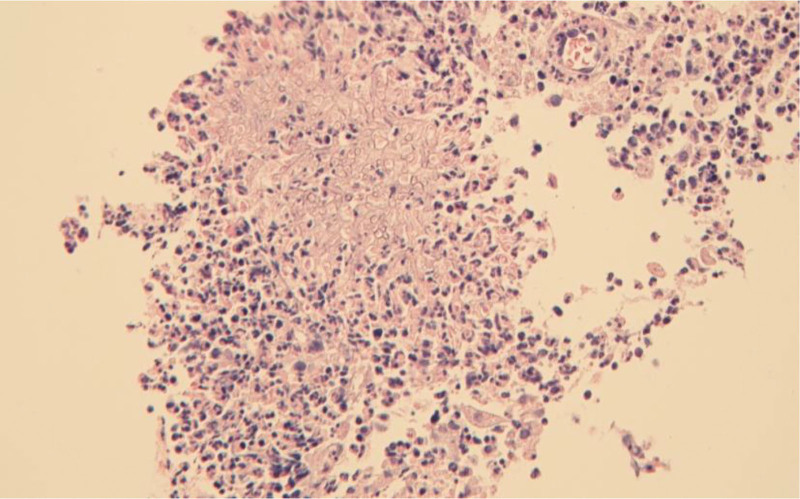
Pathological examination of the polypoid neoplasm revealed severe chronic inflammation, neovascularization, local fibrosis, and the presence of Aspergillus.

**Figure 3.**
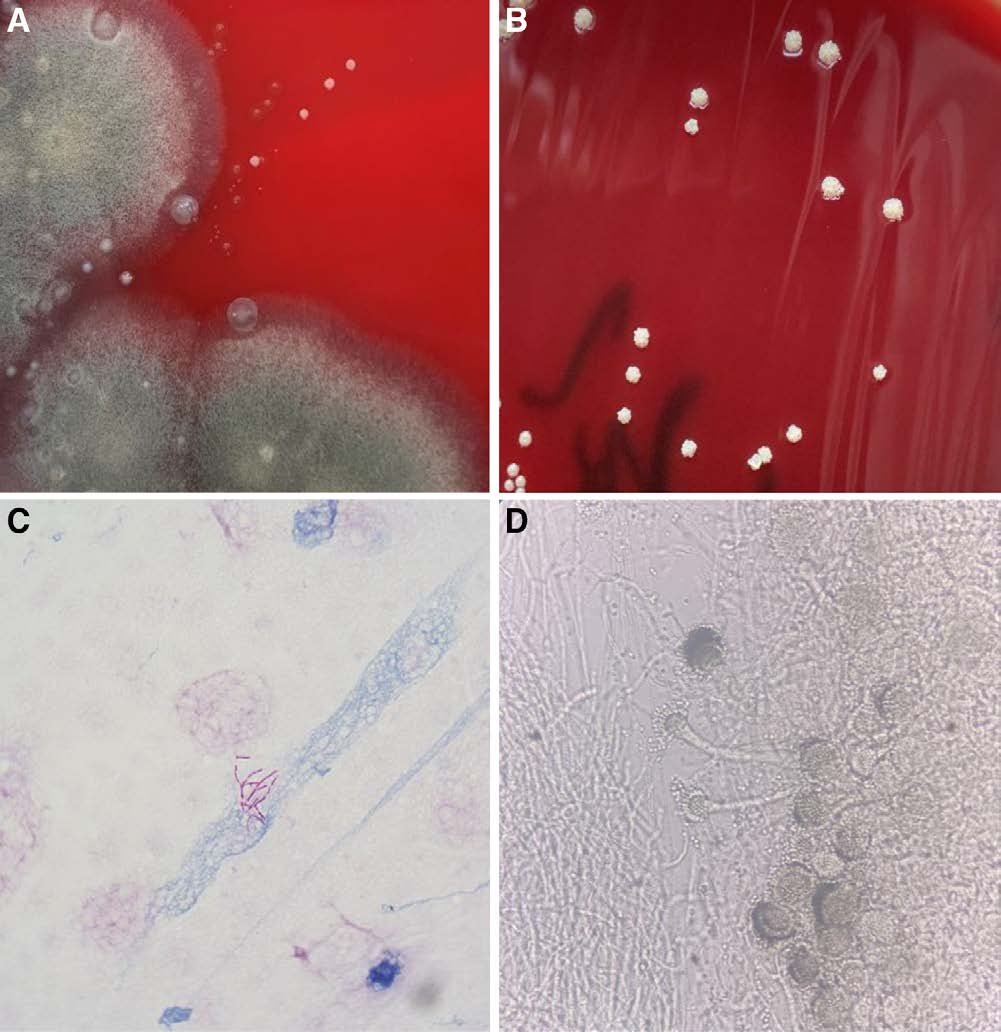
After 5 d of culturing BALF sample, Aspergillus fumigatus and Nocardia species were discovered on Columbia blood agar plates. Aspergillus fumigatus presented as cottony colonies with a greenish color, while Nocardia spp presented as millet grain size (A). Extending the cultivation time of Nocardia, the colony appears chalky, lacking luster, irregular, and wrinkled in shape, with edges trapped in the culture medium (B). Sputum samples were centrifuged and sediment was taken for acid fast staining. Under high magnification microscopy, weak positive acid fast staining was found, with slender filaments and branching Nocardia genus (C). Examination of the Aspergillus hyphal wet mount revealed flask-shaped vesicles and single-layered conidia (D). BALF = bronchoalveolar lavage fluid.

Upon admission (Day 1), a preliminary clinical diagnosis of pulmonary infection was retained, and empirical symptomatic treatment was initiated using a combination of piperacillin-sodium tazobactam and etimicin sulphate. To clarify the etiology, bronchoscopy was performed (Day 5). Bronchoscopy revealed mucosal congestion and edema in the apical segment of the right upper lobe of the lung. Filamentous substance can be seen on the surface of the bronchial. Two spherical polypoid new organisms could be seen, which obstructed the opening of subsegment of the bronchus and moved along with the respiratory activity (Fig. 1A). The pathological examination of new organisms disclosed evidence of severe chronic inflammation, accompanied by neovascularization and localized fibrosis. Notably, the presence of Aspergillus fungi was observed within the tissue (Fig. 2). Next-Generation Sequencing (NGS) was performed on bronchoalveolar lavage fluid sample (BALF) sample (Day 6). The results revealed the presence of *A fumigatus* (1 × 10^3 copies/mL) and *N cyriacigeorgica* (1 × 10^2 copies/mL). While there have been limited reports of mixed infections involving both pathogens in immunocompetent patients, it is noteworthy that our patient had a history of occupational exposure and significant risk factors for airborne infections. We promptly communicated the NGS results to the microbiology laboratory, allowing for an extended culture duration to yield more clinically meaningful pathogenic results. After 9 days of antibiotic treatment, a follow-up chest CT scan (Day 9) displayed exacerbation of patchy and nodular high-density opacities in both lungs, and the treatment regimens were considered to have poor efficacy. After 5 days of culturing BALF, *A fumigatus* and Nocardia spp were discovered on Columbia blood agar plates(Day 11). Aspergillus fumigatus presented as cottony colonies with a greenish color, while Nocardia spp presented as millet grain size (Fig. 3A). Extending the cultivation time of Nocardia, the colony appears chalky, lacking luster, irregular, and wrinkled in shape, with edges trapped in the culture medium (Fig. 3B). Sputum samples were centrifuged and sediment was taken for acid fast staining. Under high magnification microscopy, weak positive acid fast staining was found, with slender filaments and branching Nocardia genus (Fig. 3C).Examination of the Aspergillus hyphal wet mount revealed flask-shaped vesicles and single-layered conidia (Fig. 3D). In light of the patient clinical presentation, treatment course, occupational exposure history, changes observed in the chest CT scan, microbiological findings, and pathological results, we have arrived at the diagnosis of pulmonary aspergillosis and pulmonary nocardiosis in the patient.

**Figure 4.**
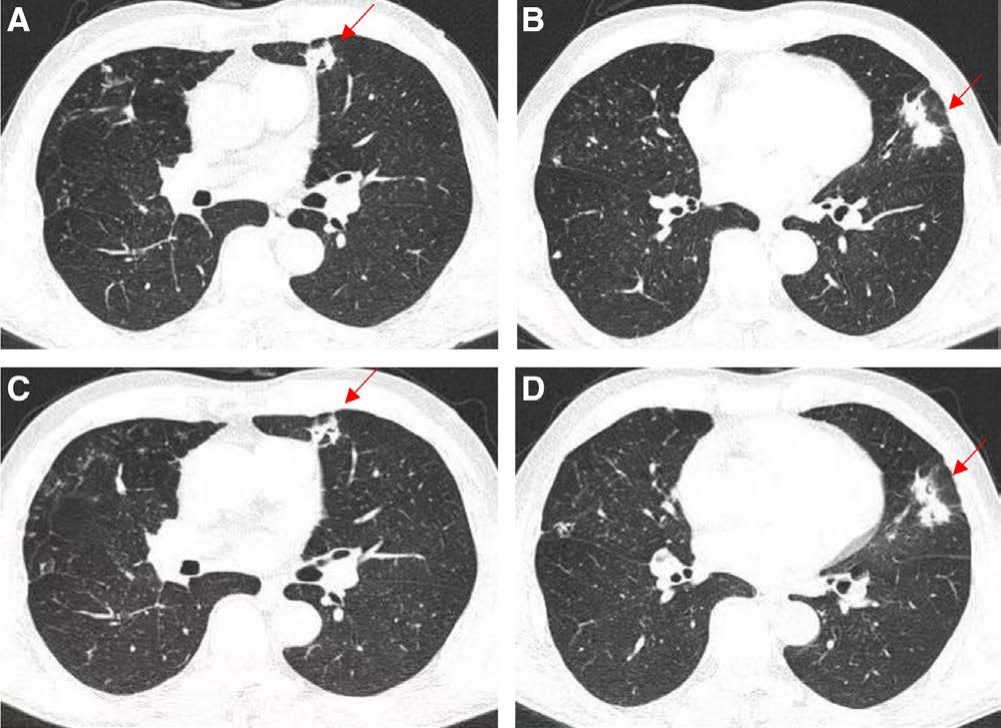
After adjusting the treatment plan to voriconazole and SMZ-TMP for 11 d, the chest CT scan (Day 20) (C, D) shows a reduction in the size of the localized lesion compared to previous antibiotic treatment (Day 9) (A, B) (indicated by the red arrows).

Based on the aforementioned diagnosis, we adjusted the treatment regimen. Voriconazole (200 mg q12 h) was administered for the treatment of the *A fumigatus* infection, while sulfamethoxazole-trimethoprim(SMZ-TMP) tablets (1 tablet qid) were employed to address the *N cyriacigeorgica* infection. After 6 days of this protocol (Day 18), the patient ESR decreased significantly (ESR 90.00 mm/h), and improvements were noted in his anemia and hypoproteinaemia (HB 116.20 g/L, ALB 37.15 g/L) compared to the values at baseline. Patient had no adverse reactions to sulfonamides, and this allowed for an increase in the dosage of SMZ-TMP tablets to 2 tablets qid. After adjusting the treatment plan to voriconazole and SMZ-TMP for 11 days, the chest CT scan (Day 20) (Fig. 4C and D) shows a reduction in the size of the localized lesion compared to previous antibiotic treatment (Day 9) (Fig. 4A and B). Bronchoscopy (Day 22) shows reduced congestion and edema in the mucosa of the opening of the apical segment of the upper lobe of the right lung, with a reduction in the filamentous material on the surface of the mucosa (Fig. 1B). After 2 weeks of treatment (Day 26), the patient body temperature normalized, with no cough or sputum, and an overall assessment indicated effective treatment. In accordance with the patient and family wishes, discharge was arranged. Following discharge, the patient continued oral administration of voriconazole and SMZ-TMP. A telephone follow-up at 6 months, the patient reported no clinical symptoms, stopped taking the medication voluntarily 1 month ago and refused to be reviewed.

## 3. Discussion

PN is a suppurative lung infection caused by aerobic Actinobacteria of the Nocardia genus. It predominantly arises from inhalation of Nocardia spores or fragmented hyphal elements through the respiratory tract, triggering suppurative inflammation and necrosis within the lungs. *N cyriacigeorgica* is filamentous growth bacilli, with gram-positive, and acid-fast staining is weakly positive, which are uncommon in clinical pathogenesis due to their slow growth rate. Therefore, prompt communication with the clinical microbiology department and extension of the incubation period are essential. Clinical manifestations of PA infections include allergic bronchopulmonary aspergillosis, chronic pulmonary aspergillosis and invasive pulmonary aspergillosis.^[1]^ The most common among these is *A fumigatus*, whose spores or hyphae infiltrate tissues upon inhalation, leading to granuloma formation and progression to invasive aspergillosis.^[2]^ PN and PA share several attributes and common risk factors. Both primarily stem from respiratory tract infections, predominantly affecting hosts with immunosuppression or underlying lung diseases. PN commonly affects immunocompromised hosts and can also occur in structural lung diseases such as cystic fibrosis and bronchiectasis.^[3]^ allergic bronchopulmonary aspergillosis occurs almost exclusively in patients with asthma or cystic fibrosis. Chronic pulmonary aspergillosis is mainly associated with patients having underlying lung diseases (post-tuberculosis sequelae, nontuberculous mycobacterial infections, lung cancer, bronchiectasis) and those with mild or no immune suppression.^[4]^ Invasive pulmonary aspergillosis is primarily observed in immunodeficient populations. Typical imaging manifestations of invasive fungal disease include nodules, masses, segmental or subsegmental solid changes, and ground glass shadows. Halo sign, anti halo sign, hypodensity sign and air crescent sign can better differentiate fungal pneumonia.^[5]^

The patient had a 40-year smoking history. The literature suggests a correlation between lung parenchymal diseases and exposure to tobacco smoke, which can lead to chronic obstructive pulmonary disease, mechanisms of interstitial damage, various pathological changes, and lung fibrosis.^[6]^ By residing in a rural area and having worked as a security guard at a valve factory over the past year, the patient was exposed to a damp environment with a detectable mouldy odor, making him susceptible to inhaling pathogenic spores or fragmented hyphae, leading to a pulmonary infection. The patient in this case exhibited typical respiratory infection symptoms, such as fever and cough. The pathological analysis of spherical polypoid, culturing and NGS of the BALF sample revealed a mixed infection involving *A fumigatus* and *N cyriacigeorgica*. Considering the patient general antibacterial therapy, chest CT imaging progression, a 40-year history of smoking, and the presence of numerous pathogens in the environment where he lived, these factors were all identified as risk factors. Treatment with voriconazole and SMZ-TMP led to clinical improvement, and the patient was discharged. A 6-month follow-up via telephone indicated complete recovery and discontinuation of treatment. A telephone follow-up at 6 months, the patient reported no clinical symptoms, stopped taking the medication voluntarily 1 month ago and refused to be reviewed.

The mortality rates for PA and PN are notably high, emphasizing the critical significance of prompt diagnosis and intervention. NGS technology, which circumvents traditional microbial culturing, allows for the ability to directly conduct high-throughput sequencing of nucleic acids from samples. This approach offers broad coverage and high sensitivity, delivering test results within 1 to 2 days. It is applicable to the identification of various pathogens and is particularly valuable in diagnosing newly emerging, complex, and mixed infections, as highlighted in cases of clinical urgency.^[7,8]^ In this instance, the patient condition presented with simultaneous detection of *A fumigatus* and *N cyriacigeorgica* in BALF, as revealed by NGS, merely 6 days into hospitalization. Given the patient immunocompetence, the possibility of colonization or contamination of the NGS results was considered. Initially, the treatment did not target these 2 pathogens. Instead, the ongoing antibiotic regimen was continued. However, upon reevaluating the CT scan on the 9th day of hospitalization, an aggravated pulmonary infection was observed. This, in conjunction with the cultivation of *A fumigatus and* Nocardia spp from patient samples in the clinical microbiology lab (culturing of Nocardia was extended to the 5th day after learning of the NGS results), prompted the initiation of a treatment regimen targeting both Aspergillus and Nocardia. Following 6 days of the new treatment approach, a decrease in ESR and a reduction in lesion size were noted. The treatment strategy was continued.

Due to the rarity of combined infections involving Aspergillus and Nocardia in immunocompetent populations, there is currently no consensus on the initial treatment selection or duration. In vitro susceptibility testing plays a pivotal role in determining the effectiveness of antimicrobial agents. This article has certain limitations. Our laboratory was unable to conduct susceptibility testing for these 2 pathogens. Consequently, the therapeutic choices were guided by previous literature reports. Regarding treatment for PA, voriconazole is a lipophilic drug that is widely distributed in lung tissue, alveoli and epithelial cell lining fluid and is the primary choice for medical treatment.^[9]^ For PN, TMP-SMX has been a longstanding preferred therapeutic option.^[10–13]^ In this particular case, a combination therapy involving voriconazole and TMP-SMX was administered, his clinical symptoms were stable, but because the patient refused to be reviewed and did not undergo a review of laboratory tests such as chest CT, we will continue to follow up and observe the patient.

Originating from a search on PubMed, a retrospective analysis was conducted on case reports, and a total of 20 instances of mixed infections involving both Aspergillus and Nocardia were found from 1984 to 2023 (Table 1). Among these, patients aged 50 and above constituted half of the cases (50%), with the majority being male (75%). Each case report in the table presented multiple predisposing factors contributing to these mixed infections and we tabulated and assessed the most significant Predisposing factors of literatures (Table 2). Revealing a predominance of immunocompromised hosts. The instances encompassed cases of immunosuppressive drug therapy,^[2,21,31]^ hematologic malignancies,^[18,24,25]^ organ transplantation,^[15,16,19,20]^ chronic granulomatous disease,^[9,14]^ and systemic lupus erythematosus,^[29]^ and a case of a tooth extraction in a mildly immunosuppressed patient.^[30]^ Such cases were also observed in nonimmunosuppressed hosts, including patients with diabetes,^[28]^ allergic disorders,^[26]^ bronchial thermoplasty therapy^[27]^ ulcerative colitis during golimumab therapy,^[23]^ and even accidental drowning incidents.^[17,22]^ Pulmonary involvement was universal across all patients, while infection was also identified in subcutaneous tissues, the lymph nodes, and intracranial and central nervous system sites. A majority (60%) of the patients demonstrated a substantial recovery posttreatment.

**Table 1:** Literature review of Co-infections involving both Aspergillus and Nocardia (from 1984 to 2023).

Reference	Country age/gender	Infection site	Outcome
Casale TB,^[14]^ 1984	USA/15/man	Pulmonary	Not available
Carter JM,^[15]^1990	USA/49/man	Pulmonary	Died
Monteforte JS,^[16]^1993	USA/63/man	Pulmonary	Recovered well
Fernández Peláez JM,^[2]^2000	Spain/39/man	Pulmonary	Died
van Dam AP,^[17]^2005	Netherlands/22/man	Pulmonary	Recovered well
Al-Anazi KA,^[18]^2008	Saudi Arabia/67/man	Pulmonary	Recovered well
Hamadani M,^[19]^2008	USA/50/woman	Pulmonary	Recovered well
Cabada MM,^[20]^2010	USA/65/man	Pulmonary	Recovered well
Misra DP,^[21]^2014	India/37/man	Pulmonary	Recovered well
Yamawaki S,^[22]^2016	Japan/84/man	Pulmonary	Died
Alonso-Sierra M,^[23]^2016	Spain/53/man	Pulmonary	Not available
Trastoy R,^[24]^2017	Spain/81/woman	Pulmonary	Not available
Dotson J,^[25]^2018	USA/64/woman	Bronchopulmonary, subcutaneous tissues, lymph nodes central nervous system	Died
Wu J,^[26]^2018	China/55/woman	Pulmonary	Recovered well
Matsubayashi S,^[27]^2018	Japan/35/woman	Pulmonary	Recovered well
Raj R,^[28]^2020	India/59/man	Pulmonary	Recovered well
Tian X,^[9]^2022	China/3/man	Pulmonary, oral maxillofacial, perianal	Died
Roy M,^[29]^2022	USA/39/man	Pulmonary, central nervous system	Recovered well
Jinlin G,^[30]^2022	China/62/man	Intracranial, pulmonary	Recovered well
Yan H,^[31]^2022	China/57/Man	Pulmonary	Recovered well

**Table 2:** Predisposing factors of literatures (from 1984 to 2023).

Reference	Predisposing factors
Casale TB,^[14]^ 1984	chronic granulomatous disease
Carter JM,^[15]^1990	Kidney transplant, immunosuppressive therapy
Monteforte JS,^[16]^1993	heart transplant
Fernández Peláez JM,^[2]^2000	Bronchiolitis obliterans with tissue pneumonia, receiving glucocorticoid therapy
van Dam AP,^[17]^2005	Near-drowning incident
Al-Anazi KA,^[18]^2008	T-Cell Lymphoma, receiving immunosuppressive therapy
Hamadani M,^[19]^2008	Immunosuppression and chronic graft-versus-host disease
Cabada MM,^[20]^2010	lung transplantation
Misra DP,^[21]^2014	Adult onset Still disease with steroid tacrolimus therapy
Yamawaki S,^[22]^2016	Drowning incident
Alonso-Sierra M,^[23]^2016	Ulcerative Colitis with Golimumab Therapy, mild chronic obstructive pulmonary disease, bronchiectasis and Hodgkin lymphoma
Trastoy R,^[24]^2017	B-cell Hodgkin lymphoma and diabetes mellitus.
Dotson J,^[25]^2018	Chronic Lymphocytic Leukaemia with Ibrutinib therapy
Wu J,^[26]^2018	Allergic bronchopulmonary aspergillosis, Repeatedly coughed up sputum for 20 y, and history of tuberculosis
Matsubayashi S,^[27]^2018	Severe asthma with Bronchial thermoplasty therapy and systemic steroids therapy
Raj R,^[28]^2020	High blood pressure, diabetes
Tian X,^[9]^2022	X-linked chronic granulomatous disease
Roy M,^[29]^2022	Systemic lupus erythematosus with hydroxychloroquine and prednisone therapy
Jinlin G,^[30]^2022	A Mildly Immunosuppressed Patient who had a Tooth Extraction
Yan H,^[31]^2022	Chronic viral hepatitis B and type 2 diabetes mellitus, high-dose glucocorticoids therapy

In our search on PubMed, we found that reports of co-infections involving *A fumigatus* and *N cyriacigeorgica* in the literature are notably scarce. Most cases have been documented in patients with compromised immune systems or underlying pulmonary conditions. In contrast, our patient did not present with any of these risk factors. Furthermore, there have been no recent incidents such as near-drowning or other accidents in the patient history. Clinical practitioners are effectively leveraging the notable advantages and value of NGS in detecting rare and mixed pathogens associated with pulmonary infections. By integrating traditional microbiological culture results and clinical manifestations, timely diagnosis and treatment were achieved, leading to the successful recovery of the patient. This case report holds significant educational and research value, aiming to enhance clinical awareness of such rare bacterial mixed pulmonary infections.

## Author contributions

**Conceptualization:** Jiaqing Ye, Yahua Li, Lijie Zhang.

**Investigation:** Jiaqing Ye, Jiahao Hao, Minghui Song.

**Methodology:** Jiaqing Ye, Yahua Li, Lijie Zhang.

**Project administration:** Cuiying Zheng, Zhongjun Feng.

**Resources:** Yahua Li, Lijie Zhang.

**Supervision:** Cuiying Zheng, Yinqi Huang.

**Validation:** Yumei Guo, Weili Gao.

**Visualization:** Yumei Guo, Weili Gao.

**Writing – review & editing:** Yahua Li, Lijie Zhang.

**Writing – original draft:** Jiahao Hao, Minghui Song.
